# Sanguinaria Canandensis

**Published:** 1876

**Authors:** L. B. Anderson

**Affiliations:** Hanover County, Virginia


### SANGUINARIA CANADENSIS.

BY L. B. ANDERSON, HANOVER COUNTY, VIRGINIA.

I know of no article in the Materia Medica which has stood so high at one period, and has gone so much into disuse, as this. Nor do I know of any agent, the therapeutical effects of which, have been more fully appreciated, and more correctly described in some respects, and so little understood, and so grossly misrepresented in others. At one period of its history, it was esteemed almost a specific in croup and asthma, besides exerting highly sanative effects in rheumatism, jaundice, diarrhea, pneumonia, and bronchitis. While some of its advocates gave it in almost every disease to which our flesh is heir, others speak of it as a "violent, acrid and narcotic emetic."

This contrariety of opinion has doubtless resulted from the character and preparation of the article, used by different practitioners. I have been in the daily habit of using blood root, in some of its preparations, for more than fifteen years, and feel satisfied that I have been enabled to determine, with great precision and certainty, its therapeutical effects and medical value. And I think there is but little doubt, that those who have spoken in the highest terms of praise of its virtues, as well as those who ascribed to it acrid properties, have spoken equally true.

Sanguinaria may be gathered at any period of the year when it can be found, and I have found but little difference in its strength or properties, no matter when collected. The first, but one, of the delicate and beautiful flowers which make their appearance in the early spring—it is more easily found at that time than any other. But when it has been well located it can be easily found in mid-winter, by scraping away the leaves and mould, when the white pulp capping the root may be easily found. I think the very best preparation I have ever made of it was derived from the root gathered by a brave Confederate soldier, in January 1864. I had employed him to collect it for me during the other seasons, when it could be easily found by the bloom or leaves, and desiring to obtain a fresh supply, wrote to his commanding officer, who was then in winter quarters, to permit him to visit me for the purpose specified. He was promptly sent, and in a few hours after his arrival he had collected several pounds. The usual time for collecting it for market is in the early spring, when there is more sap, and hence less power in a given quantity than at any other season.

When recent, sanguinaria is the mildest, safest and most reliable emetic for young children I have ever used. That is, when given in tincture or syrup. I can't imagine why the powdered root should ever have been given internally. For it is exceedingly acrid, and possesses great power in destroying fungous growths. Hence it is often used for fistulos in horses, and cancers in the human subject. And I can well imagine, that all which has been said of its acrid emetic properties, may be true of the powdered root; but in tincture or syrup it produces emesis with but little nausea, and there is never any retching after the first vomit. This is not literally true of the old root, or the tincture which has been kept for a length of time. A tincture or syrup made of the old root contains an acrid principle, when first made, somewhat similar to the powder. But that made from the recent root is mild and certain in its effects. Nor will a tincture made of the best root, retain its properties for many months. I never rely on it, when it has stood even three months. After a few weeks, there is a deposit on the bottle, which I have never been able to remove, and the fluid loses its blood-red appearance, and most of its potency. I have endeavored to make a preparation which would preserve it for a much longer time, and think I have retained all the properties of the recent root for at least a year without deterioration. This has been effected by making a syrup of such strength as to make a teaspoonful equivalent to twenty drops, the usual dose of the tincture, made by mascerating four ounces of the root in one quart of diluted alcohol. The syrup is not objectionable to children—though it has a disagreeable taste—for there is nothing nauseating in it. A child may be made to vomit with ipecac, and the smallest quantity will nauseate him. Not so, however, with blood-root.

Its most marked and valuable effects are manifested in the mucous membranes of the fauces, throat, larynx, trachia, and bronchia; producing a copious secretion and flow of mucous. And the beneficial effects I have witnessed from its use in phthisis, I suppose, result indirectly from this. While it exerts this specific effect upon the mucous lining of the air passages, I have seen very decided impressions made upon the mucous coats of the stomach and bowels. In chronic gastritis and typhoid fever, when the tongue was red and dry, I have used it with most encouraging results, in combination with chlorate of potash. I have also seen it exert very happy effects in diarrhea in children five or more years of age, resulting from the use, and consequent irritation of unripe fruit. In the vulgarly called "spring sickness" of children, attended with nausea, debility, bloated features and a tallow complexion, a few doses given in the early morning so as to produce free emesis, I have often seen to produce entire relief. And while in ordinary catarrhs, influenzas, etc., I would not care to have any other agent, there are three diseases for which I esteem it above all price. They are—

1. Croup. The syrup, given in the early stages, in full doses, every fifteen minutes, until it produces free vomiting, and continued thereafter every hour, more or less, according to the severity of the symptoms, has given me more satisfaction than any other agent I have ever used. One remarkable difference between this and most other emetic agents is, that it does not produce such copious perspiration as do others. Hence, it is much less liable to predispose to relapses in croup or catarrh. Sometimes it may be necessary to push the emesis to several repetitions when the symptoms are grave. But, I seldom wait long after the second free vomiting, if there is not a marked improvement, before giving a large dose of calomel, and continuing the blood-root in smaller doses and longer intervals. I have not seen more than two cases in several years, in which I needed to use any other agent in croup.

2. Asthma. I have never seen any agent afford such marked and grateful relief in a violent attack of asthma as this. I give it, as in croup, until vomiting or relief occurs. It sometimes fails, but the failure has been a rare exception in my experience. I remember being called to see Dr. A. E. Peticolas in 1862, while laboring under a violent paroxysm of this distressing complaint. I had a beautiful preparation just made from best recently dried root, and proposed to give him a dose. He objected to taking it, unless I would tell him what it was, and why I wished to give it. I persisted in withholding the name and effect I hoped to produce; and the paroxysm becoming so violent, he drank it down, and in a few minutes vomited freely. A copious stream of tenacious mucous poured for several minutes from his lungs, affording prompt, and for the time, entire relief. He assured me that he had never experienced such decided relief in so short a time. I was consulted by a gentleman from Washington City, who was subject to violent attacks of asthma, from which he was never relieved under three weeks. I gave him the blood-root, and in three days he was entirely relieved. I continued to supply him with it, giving him twenty drops of the tincture in a wineglass of sweetened water, three times a day for several months, which effected a perfect cure.

3. Diphtheria. I have seen several cases of this disease ushered in with violent symptoms--high fever, sore throat, headache, etc.—relieved in a few hours by giving emetic doses of blood-root, within a few hours of the outset of the disease. When the disease has lasted for several hours, I generally give calomel, oil and turpentine to evacuate the bowels, apply kerosene oil and flannel to the throat, and give the tincture of sanguinaria in a solution, sweetened, of small doses of chlorate of potash, every three hours. If the patient can use a gargle, I direct the throat to be gargled very gently several times a day, with a solution of ʒ i of carbolic acid to one quart of water. I prohibit the use of all masses and irritating applications. Since I have adopted this method of treatment, either from the milder form of the disease, or the remarkable efficacy of the remedies, I have been blessed with the happiest results. I have every reason to attribute the success to the latter cause.

